# Preclinical and Clinical Evidence of Immune Responses Triggered in Oncologic Photodynamic Therapy: Clinical Recommendations

**DOI:** 10.3390/jcm9020333

**Published:** 2020-01-24

**Authors:** Irati Beltrán Hernández, Yingxin Yu, Ferry Ossendorp, Mladen Korbelik, Sabrina Oliveira

**Affiliations:** 1 Pharmaceutics, Department of Pharmaceutical Sciences, Faculty of Science, Utrecht University, 3584 CG Utrecht, The Netherlands; i.beltranhernandez@uu.nl; 2 Division of Cell Biology, Department of Biology, Faculty of Science, Utrecht University, 3584 CH Utrecht, The Netherlands; y.yu@uu.nl; 3 Department of Immunohematology and Blood Transfusion, Leiden University Medical Center, 2333 ZA Leiden, The Netherlands; f.a.ossendorp@lumc.nl; 4 Department of Integrative Oncology, BC Cancer, Vancouver, BC V5Z 1L3, Canada; mkorbelik@bccrc.ca

**Keywords:** photodynamic therapy, immunogenic cell death, innate and adaptive immunity, immunomodulation

## Abstract

Photodynamic therapy (PDT) is an anticancer strategy utilizing light-mediated activation of a photosensitizer (PS) which has accumulated in tumor and/or surrounding vasculature. Upon activation, the PS mediates tumor destruction through the generation of reactive oxygen species and tumor-associated vasculature damage, generally resulting in high tumor cure rates. In addition, a PDT-induced immune response against the tumor has been documented in several studies. However, some contradictory results have been reported as well. With the aim of improving the understanding and awareness of the immunological events triggered by PDT, this review focuses on the immunological effects post-PDT, described in preclinical and clinical studies. The reviewed preclinical evidence indicates that PDT is able to elicit a local inflammatory response in the treated site, which can develop into systemic antitumor immunity, providing long-term tumor growth control. Nevertheless, this aspect of PDT has barely been explored in clinical studies. It is clear that further understanding of these events can impact the design of more potent PDT treatments. Based on the available preclinical knowledge, recommendations are given to guide future clinical research to gain valuable information on the immune response induced by PDT. Such insights directly obtained from cancer patients can only improve the success of PDT treatment, either alone or in combination with immunomodulatory approaches.

## 1. Introduction

Photodynamic therapy (PDT) is a therapeutic procedure that has proven successful for the treatment of cancer in the clinic [1], as well as non-oncologic indications [2,3]. PDT is approved for the treatment of some skin and organ cancers, such as bladder cancer, squamous cell carcinoma of the head and neck, and esophageal cancer. In addition, numerous clinical trials continue evaluating the use of oncologic PDT [1,4], highlighting the promise of this therapy.

PDT involves the administration of a light-activatable molecule or photosensitizer (PS), which accumulates at the tumor area, driven by the enhanced permeability and retention effect (EPR), and subsequent local illumination of the tumor tissue to excite the PS. This triggers photochemical reactions that generate numerous highly reactive oxygen species (ROS), eventually leading to direct cytotoxic effects on the tumor cells. Of note, PDT protocols involving a short time interval between PS administration and light application mainly target the tumor vasculature and, in this context, the tumor cell death is largely attributed to vascular occlusion, an approach known as vascular PDT [5]. In either case, damage to surrounding normal tissues is limited and this strategy is thus considered to be selective to the treated tumor site [6], especially when compared with other conventional anticancer strategies.

**Figure 1:**
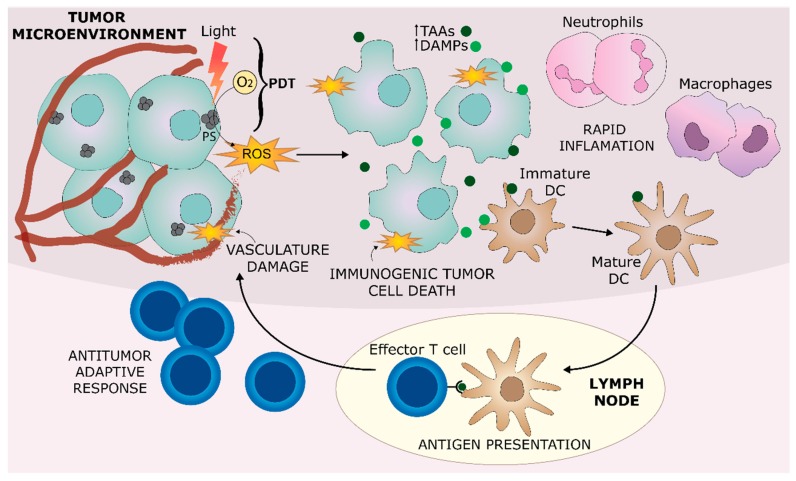
Overview of the antitumor mechanisms of PDT. PDT combines light, oxygen, and a photosensitizer (PS) resulting in the generation of reactive oxygen species (ROS) within tumor cells and tumor-associated vasculature. This leads to a direct cytotoxic effect in tumor cells as well as vasculature shutdown, which in turn results in tumor death due to starvation. This initial tumor destruction triggers a rapid localized inflammation at the tumor site consisting mostly of neutrophils and macrophages. ROS induce an immunogenic tumor cell death (mainly apoptosis and necrosis) that involves exposure/release of damage-associated molecular patterns (DAMPs) from dying cells. Antigen presenting cells (mainly dendritic cells) will be stimulated by these DAMPs, engulf tumor associated antigens (TAAs), and present antigenic peptides to effector T cells, thereby orchestrating an antitumor adaptive response, which could provide systemic tumor immune control in the long term.

Preclinical studies describe three different but interrelated mechanisms that contribute to the tumor growth control and occasional complete tumor destruction observed after PDT treatment [7,8] (Figure 1). First, PDT-generated ROS directly kill malignant cells at the primary site, via mainly apoptosis, necrosis and/or autophagy. Secondly, PDT damages the endothelial cells of tumor-associated vasculature, resulting in a significant decrease in blood flow that can lead to tumor death due to starvation. These two mechanisms are responsible for the initial tumor ablation, which appears to trigger an early localized inflammatory response that constitutes the third antitumor mechanism. This early response can activate the immune system and thereby facilitate the clearance of remaining tumor cells in the treated site. In a later phase, an adaptive immune memory may develop as well, leading to a systemic response, capable of preventing tumor recurrence and the formation of tumor metastases, in the long term. Immunostimulation in this context appears to be dependent on the two other antitumor mechanisms, i.e., the type of tumor cell death mediated by ROS and the extent of the tumor-associated vasculature damage.

Tumor cells benefit from a dysfunctional immune environment incapable of eliciting an antitumor response partly due to the absence of factors that can stimulate innate immune cells [9]. PDT seems to overcome this dysfunction by induction of immunogenic tumor cell death pathways, mainly immunogenic apoptosis and necrosis. An innate immune response can be triggered by the exposure or release of danger signals from dying and damaged cells, so-called damage-associated molecular patterns (DAMPs) [9,10]. These DAMPS alone or in association with tumor antigens can be recognized by antigen presenting cells (APCs), which may result in the development of an adaptive immune response against the tumor [10,11,12] (Figure 1).

PDT is now established as a clinical treatment for some cancers and non-malignant diseases, but it is still underutilized in the clinic and it has not yet reached its full potential. Although antitumor immunity after PDT has been reported in animal tumor models, the role of the immune system in the therapeutic outcome of clinical PDT is still unclear. Of note, studies use a wide range of PDT protocols that include different PS, doses, tumor models, and illumination conditions. All these factors can affect the development of antitumor immunity and treatment outcome, making it challenging to compare studies side by side and draw solid conclusions.

In this review, substantial evidence of the immunostimulatory effects of PDT is provided. For this, we begin by describing the possible mechanism of how PDT induces immune responses, followed by the evidence of immune responses post-PDT, from preclinical studies (particularly using the PS aminolevulinic acid (ALA) or Photofrin), to the available clinical studies reporting on these effects. Special attention is also given to combined strategies described at the preclinical level to exploit the immunobiology of PDT for more potent and prolonged responses. Lastly, recommendations are given for future clinical trials to collect additional information on the evidence of immune responses, and critical points for the translation of the available preclinical knowledge into the clinic are also discussed here. Importantly, when the recommendations given are implemented in new clinical trials, significant and unique information will be obtained, which is expected to contribute to a better understanding of PDT effects and to improve the success of treatment.

## 2. How PDT Induces Immune Responses against the Treated Tumor

The critical event for the development of PDT-induced antitumor response is the engagement of cellular stress signaling networks caused by the infliction of oxidative stress in targeted cancer cells, that creates a threat of proteostasis impairment [13]. These evolutionarily well-preserved canonic protection mechanisms are based on the elaborate and universal stress response signaling mediated by a network of harmonized signal transduction pathways [14]. The means for controlling tissue homeostasis at the PDT-treated site includes molecular repair mechanisms for recovery of stressed cells, local inflammatory responses, elimination of impaired tissue components and of damaged cells. The latter encompasses not only programmed cell death mechanisms (apoptosis, necroptosis, pyroptosis, autophagy, and others), but also immune rejection [13].

Stress signal transduction pathways are usually triggered in cells of PDT-treated tumors by sensors (kinases or transcription elements) released from repressed state by the presence of stressors, mostly proteins becoming unfolded/misfolded due to the photooxidative damage. Such sensors reportedly involved in response to PDT include inositol-requiring element-1 (IRE1), activating factor 6 (ATF6), protein kinase R-like ER kinase (PERK), heme regulator inhibitor kinase (HRI) from unfolded protein response (UPR), and integrated stress response (ISR) pathways [13]. These sensor kinases phosphorylate, upon activation, their downstream responders, which are nuclear transcription factors that can now translocate from cell cytoplasm into nucleus and bind to stress response elements located in the promoters of targeted genes [15]. This leads to the expression of certain genes encoding stress-relieving proteins dedicated to the maintenance of proteostasis, which range from chaperons, other proteins that become DAMPs, antioxidant proteins, to factors promoting cell death [13]. Stress signaling mediated by PERK and IRE1 controls the activities of NF-κB and Toll-like receptors (TLRs) that are recognized as critically important in PDT-induced host responses [11,16,17]. Various stress signaling pathways interact at multiple points with signals regulating innate and adaptive immune activity and immunogenic cell death development, as well as controlling the activity of regulatory immune cells [13,17]. Another important circumstance is that stressed cells become highly immunogenic [18]. This is mainly due to cryptic translation events triggered by stress signaling-mediated accrual of alternate initiation factors capable of translating normally untranslated RNA regions [19], which results in the expression of neoantigens that are not controlled by immunotolerance mechanisms. In such a way, PDT can elicit a strong immune response against treated tumors, which appears to share features with other rapid tumor ablating modalities induced cellular stress response, including those based on thermal effects, high hydrostatic pressure treatment, and electric effect [20,21].

## 3. Preclinical Evidence of Antitumor Immunity Induced by PDT

Here, we first describe the effect of PDT on the innate immunity, followed by that on adaptive immunity, particularly focusing on the PS Photofrin and ALA. This choice of PSs is based on the fact that long-established PDT protocols are used for these PSs, hereby minimizing the influence that the PDT protocol can have on the subsequent immunomodulation. Furthermore, both PSs are two of the few extensively investigated in animal studies and the ones mostly investigated in cancer patients (even if in few studies in total), thus easily enabling correlation of data and translation of knowledge into the clinic. A summary of the main characteristics and aspects concerning Photofrin and ALA are presented in Table 1.

### 3.1. PDT and the Innate Immune System

#### 3.1.1. Neutrophils

Being the most numerous leukocytes in human body, neutrophils are capable of causing damage at inflammatory sites, and influencing other immune cell functions. Rapid and massive increase of neutrophils is one of the manifestations in PDT-mediated acute inflammation. However, this notion is mainly derived from studies using Photofrin. Within 5 min, high numbers of neutrophils can already be seen at the treated tumor site [22], and this lasts until 24 h after PDT. More recently, a noninvasive manner has been developed to monitor neutrophil activation at the tumor site using in vivo imaging of luminol chemiluminescence [23]. Of note, pronounced neutrophilia in blood was also observed in mice treated with Photofrin-PDT, which is composed of an early wave of elevation in blood neutrophil levels during the initial hours (3–4 h), followed by an even more pronounced increase around 8–10 h after PDT [24,25,26]. Such surge is presumably an accelerated generation and mobilization of neutrophils from the bone marrow [26], promoted by IL-1 and Granulocyte colony-stimulating factor (G-CSF) that are known to be induced by PDT [27]. In ALA-PDT, these two waves of neutrophilia have not been characterized. A dramatic increase of neutrophils in blood and at the tumor site in rats has been described after systemic ALA-PDT [28].

The infiltration of neutrophils into the tumor sites is thought to be mediated by the intercellular adhesion molecule of ICAM-1 [29,30] and L-selectin [25,31] expressed on the tumor vascular endothelium, as well as ICAM-1 ligands (CD11b/CD18 and CD11c/CD18) that are upregulated on neutrophils following Photofrin-PDT [30]. Although KC (mouse analog of human CXCL1) and CXCL2 are reported as major chemoattractants for the tumor infiltration of neutrophils after HPPH (2-[1-hexyloxyethyl]-2-devinyl pyropheophorbide-a)-PDT [32], these molecules have not been described in Photofrin-PDT. Besides, vasodilatation and increased vascular permeability were observed after ALA-PDT [33], which may contribute to the increased infiltration of immune cells into the tumor.

Generation of neutrophil responses has been suggested to be particularly important in the case of Photofrin-PDT. This has been reported using immunocompetent and neutrophemic rats, where the success of Photofrin-PDT depends on the number of circulating neutrophils [34]. In addition, in a recent study using a mouse model, stronger activation of neutrophils at the tumor site was shown to be prognostic for a complete response after Photofrin-PDT in the long term. However, the role of neutrophils in ALA-PDT seems less crucial than in Photofrin-PDT. Using neutrophemic rats, it has been demonstrated that depletion of neutrophils did not significantly change the cure rates of ALA-PDT [28]. It is important to note that these two studies had the same tumor animal model, PS administration route, and time interval between PS administration and light application [28,34]; however, they differ in size of the treated tumors, induction method of neutropenia, and illumination regimens, which may, at least partly, explain this discrepancy.

#### 3.1.2. Complement

Since no characterization of the complement response in animals has yet been provided in the case of ALA-PDT, this section mainly discusses studies using Photofrin. The complement system is involved in the initial recognition of tumor damage, recruitment of immune cells to the tumor site, removal of damaged/dead cells, and direct tumor lysis [35]. In fact, systemic and local complement activation has been observed following Photofrin-PDT treatment of solid tumors. Complement protein C3 has been identified as an essential chemoattractant for the advanced inflammatory infiltration [25,35] and, to a much lesser extent, for the early phase as well [25]. Blockage of C3a receptor significantly inhibits the advanced phase of neutrophilia (which appears 8 h after PDT) [24], illustrating the role of C3 in propagating the advanced neutrophilia. The rise of C3 protein in serum, however, occurs at the post-PDT time period when the neutrophilia is largely resolved [26]. Actually, C3 serum levels even temporarily drop shortly after PDT, but reach their peak at 24–72 h, and remain elevated within the first week [26,36]. The initial decline can, most likely, be explained by the elevated consumption of C3. Indeed, the activation of complement, as indicated by erythrocyte hemolysis, correlates with the time kinetics of PDT-induced neutrophilia. In addition, upregulation of C3a receptor on peripheral neutrophils and monocytes was observed at 8 h after PDT. The complement system can be activated by antibody complexes, so called classical pathway, or by damaged tissue and altered cell surfaces via the alternative pathway [35]. PDT-induced complement activation has been suggested to occur via the alternative pathway, since neutrophilia is also observed in severe combined immunodeficient (SCID) mice after PDT, which are deficient in T and B cells.

The membrane attack complex (MAC), assembled by the complement proteins C5b-9, has been observed on the cell membrane of both tumor and endothelial cells as early as 30 min post-PDT [29]. Interestingly, this lytic pore can also act as leukocyte chemoattractant. Furthermore, tumor cells after PDT are more vulnerable to complement attack, partly because of the downregulated expression of membrane-bound complement regulatory proteins (mCRPs), which prevent complement attack, on the surface of PDT-treated tumor cells [37].

#### 3.1.3. Immunogenic Cell Death

Physiological apoptosis is regarded as a tolerogenic mode of cell death in immunological terms. More recently, two morphologically equivalent but immunologically distinct subclasses of apoptosis, i.e., immunogenic and non-immunogenic apoptosis, were described giving rise to the new concept of immunogenic cell death (ICD) [38,39]. It is a form of cell death eliciting activation of the immune response in a manner similar to pathogen-infected cells [40]. ICD is characterized by exposure or release of DAMPs, such as calreticulin (CRT), high mobility group box 1 (HMGB1), heat shock protein (HSP)-70/90, and ATP. Through binding to pattern-recognition receptors (PRRs), e.g., TLRs, these DAMPs elicit activation of immune responses as endogenous analogs to pathogen-associated molecular patterns (PAMPs). For instance, ICD induces a chemokine release profile capable of recruiting neutrophils, which can eliminate residual viable cells, in a similar fashion as upon pathogenic incursion [40]. ICD is not unique to apoptosis [41]. Cell death through necrosis, necroptosis, pyroptosis, or autophagy can also induce immunogenicity. It has been shown that ICD depends on the generation of ROS and the phosphorylation of eukaryotic initiation factor α (eIF2α), which is one of the three arms of the ER stress response [41,42]. PDT can induce large amount of ROS production inside the cancer cells, thereby causing oxidative stress-based cell death. In other words, PDT may turn “cold” tumors into “hot” tumors by inducing ICD. Being known as a strong inducer of ROS-based ER stress, Hypercin-PDT is the first PDT identified as being an ICD inducer [43]. In fact, the capacity of Photofrin- or ALA-PDT to induce tumor ICD has also been explored. In particular, Photofrin-PDT treated tumors exhibit a marked increase of the surface HSP70, as well as a slight increase of chaperone GPR78, at 16 h post-PDT [11]. In another study, Korbelik et al. showed that, shortly after Photofrin-PDT (at 1 h), cell surface expression of CRT already increases on tumors, and HMGB1 rises in serum [44]. Application of topical ALA-PDT increases expression of several other DAMPs (e.g., CRT, HSP70, and HMGB1) in tumors within the first 9 h, leading to subsequent maturation of dendritic cells [45].

It is important to note that ICD is a prerequisite for induction of effective immune responses against cancer [46]. A “gold standard” protocol for the in vivo assessment of ICD in mice relies on vaccination with treated cancer cells, followed by re-challenge with living cancer of the same type in syngeneic immunocompetent animals [47]. Gollnick et al. were the first to demonstrate that Photofrin-PDT-generated tumor cell lysates are immunogenic and can be used as an anticancer vaccine. Moreover, these PDT-based cancer vaccines are significantly more protective than other ways of generating cancer vaccines (UV, freeze/thaw, or ionizing irradiation) [48]. Vaccination with Photofrin-PDT treated tumor cells induces DC maturation, increases IFN-γ and TNF-α secretion by splenocytes, as well as their cytolytic activity [48,49], and is effective against poorly immunogenic tumors [50]. The systemic tumor-specific immune response has also been reported in the case of ALA [45], where a complete and strong tumor protection is seen in challenged mice. Likewise, vaccination with DCs stimulated by ALA-PDT treated tumor cells is more potent and effective than the one obtained with DCs co-cultured with tumor cells treated with freeze/thaw [12]. Together, these data provide evidence for the capacity of PDT being a strong ICD inducer and a promising strategy for the use of PDT in the generation of cancer vaccines.

#### 3.1.4. Dendritic Cells

Being a major subpopulation of antigen presenting cells, dendritic cells (DC) phagocytize damaged tumor cells and process their antigens and migrate to local lymph nodes, where in optimal environment they could present antigens to naïve T cells, leading to subsequent T cell proliferation and activation. PDT treatment influences this process in many ways. For instance, efficient phagocytosis of dead tumor cells by DC after Photofrin-PDT [10] increased number of IL-12 expressing CD11c^+^ DCs in the tumor draining lymph nodes at 24 h after Photofrin-PDT [51], and their enhanced capacity to stimulate T cell proliferation and IFN-ƴ release [51]. Similarly, accumulation of DCs was also observed at the tumor site at 24 h after ALA-PDT [52]. Of note, immature DC are poor T-cell stimulators, which rather tend to induce specific immune tolerance [53]. Hence, DC maturation is a critical step in the induction of the immune response. In fact, accumulating evidence from Photofrin- or ALA-PDT supports that PDT-treated tumor lysates enhance DC maturation in vitro, as indicated by their enhanced expression of surface CD86, CD80, and MHCII, as well as IL-12 release [45,48,54]. This is most likely induced by the accumulation of DAMPs at the tumor site, yet further studies are needed to elucidate the exact mechanism.

In addition, other innate components such as mast cells and macrophages have been addressed in the PDT-induced innate immune response, although to a lesser extent. Increased numbers of these cells were seen at the tumor site [22] or peritoneally [24] within 24 h after Photofrin-PDT. Remarkably, it appears that NK cells contribute largely to the cure rates of tumor-bearing SCID mice after Photofrin-PDT [55], but such effect was not observed in the immunocompetent host. In conclusion, preclinical studies have provided consistent and detailed insights into the innate immune responses induced by Photofrin-PDT, such as neutrophilia, complement activation, exposure of DAMPs and increased DC activity (a number of these studies have been summarized in Table 2). Characterization of these responses is however less defined in ALA-PDT that is often applied topically for treating superficial cancer. Distinct microcirculatory effects have been described between systemic and topical application of ALA [33]. However, it is unknown if the application route could influence the host immune responses, since data were not provided in these studies [33,52].

### 3.2. PDT and the Adaptive Immune System

The key role of adaptive immunity in PDT-induced antitumor immunity was first addressed by Korbelik et al., who described that Photofrin-PDT can initiate tumor cell killing in both immunocompetent and immunodeficient mice (SCID or nude mice), whereas no long-term cure of tumors was observed in the latter [56]. The same group further demonstrated that adoptive transfer of splenocytes from the immunocompetent mice cured by PDT into SCID mice fully restored the curative effect of PDT. These adoptive splenocytes are antigen-specific, since the tumor rejection occurs only when mice were inoculated with tumors of the same origin [55]. Interestingly, such adoptive transfer obtained from tumor-bearing mice cured using X-rays were much less effective [55], suggesting a stronger immunogenic impact of tumor cell death triggered by PDT.

Evidence points to CD8^+^ T lymphocytes as the main player in PDT-mediated antitumor immune response. This is manifested by the significantly reduced or even abrogated curative effect of PDT upon selective CD8^+^ T cell depletion [55,57]. In some cases, the induced antitumor immune response is systemic, and therefore it can also be potent outside the primary tumor area. Kabingu et al. [58] described regression of distant lung tumors after local treatment of subcutaneous tumors with Photofrin-PDT. Such systemic control of tumors was accompanied by an increased cytotoxicity of splenocytes against the tumor and infiltration of CD8^+^ T lymphocytes in the untreated tumor. CD8^+^ depletion abolished the systemic effect of PDT to control distant tumors outside the primary tumor area. Interestingly, the effect of CD8^+^ cells appears to be dependent on the presence of NK cells, rather than CD4^+^ T lymphocytes [58]. In addition, a significant increase of memory T lymphocytes (CD44^hi^CD45RB^low^) was detected in the lymph nodes of mice treated with Photofrin-PDT [31], indicating the development of immune memory. Wachowska et al. showed an increased production of IFN-γ in both CD4^+^ and CD8^+^ T lymphocytes isolated from the lymph nodes, as well as splenic CD8^+^ T lymphocytes, of mice treated with Photofrin-PDT. Moreover, these CD8^+^ T lymphocytes appear to be highly cytolytic, as indicated by their increased expression of the cytolytic marker CD107 [59]. In contrast, not much information about the ALA-PDT induced adaptive immunity is available. A study has shown that memory T cells (CD44^hi^CD62L^hi^) accumulate in the tumor and spleen of ALA-PDT treated mice [60].

**Table 2:** Preclinical studies on immune responses to Photofrin- or ALA-PDT in cancer treatment.

Time Phase (after PDT)	Location	Major Immune Events	Tumor Cell Line/Model	Strain/Species	PS/Dose/Route	Illumination Protocol	Ref
Within 24 h	Tumor	-Localized neutrophil function increases at 1 h, and then decreases at 4 h -Increased influx of neutrophils at 24 h	AB12 mesothelioma	Balb/c mice	Photofrin 5 mg/kg i.v.	135 J/cm^2^ 75 mW/cm^2^	[23]
	Tumor	-Increase of neutrophils within 5 min -Increase of mast cells and other myeloid cells during 0–2 h -Increased cytotoxicity of tumor-associated macrophages at 2 h	SCCVII squamous cell carcinoma	C3H/HeN mice	Photofrin 25 mg/kg i.v.	60 J/cm^2^ 45 mW/cm^2^	[22]
	Tumor	-Increase of neutrophils within 24 h -Increased release of myeloperoxidase at 24 h, which lasts at least 4 days	Rhabdomyosarcoma	WAG/Rij rats	ALA 200 mg/kg i.v.	100 J/cm^2^ 100 mW/cm^2^	[28]
	Tumor	-Increased expression of TNF- α at 24 h	UVB induced squamous cell carcinoma	SKH-1 mice	Topical ALA, 8% cream	Multiple 30 J/cm^2^ 20 mW/cm^2^	[52]
	Peripheral blood; Peritoneal cells	-Increase of neutrophils within 24 h which is partly mediated by complement C3a -Increase of monocytes and B cells at 8 h -Increase of mast cells and macrophage in peritoneal at 8 h	FsaR fibrosarcoma	C3H/HeN mice	Photofrin, 10 mg/kg, i.v.	150 J/cm^2^ 100 mW/cm^2^	[24]
	Peripheral blood	-Increase of neutrophils within 24 h	Rhabdomyosarcoma	WAG/Rij rats	ALA 200 mg/kg i.v.	100 J/cm^2^ 100 mW/cm^2^	[28]
	Tumor draining lymph node	-Increase of IL-12 expressing APC at 24 h -Increased cross-activation of T cells by APC at 24 h	EMT6, mammary sarcoma; CT26, colon carcinoma	BALB/C	Photofrin 5 mg/kg i.v.	135 J/cm^2^ 75 mW/cm^2^	[51]
1 week	Tumor tissue	-Increase of DC, CD4^+^, and CD8^+^ T cells at 7 days	UVB induced squamous cell carcinoma	SKH-1 mice	Topical ALA, 8% cream	Multiple 30 J/cm^2^ 20 mW/cm^2^	[52]
	Tumor tissue	-Infiltration CD4^+^/CD8^+^ T cells at 7 days	PECA squamous cell carcinoma	SKH-1 mice	ALA 0.5 mM (in vitro killing for the production of cancer vaccine)	0.5 J/cm^2^ 10 mW/cm^2^	[61]
>1 week	Lymph nodes; Spleen	-Increased production of IFN- by CD4^+^ and CD8^+^ T cells in lymph nodes at 2 weeks -Increased production of IFN^−^ by CD8^+^ T cells in spleen at 2 weeks -Upregulated CD107 (marker for cytolytic activity) in splenocytes at 2 weeks	EMT6 mammary sarcoma	BALB/C	Photofrin, 10 mg/kg, i.p.	65 J/cm^2^, 47 mW/cm^2^	[59]
No specified time point available	Tumor tissue; Spleen; Lymph node; Serum	-Inhibited tumor metastases -Reduced growth of tumor re-challenge -CD4^+^CD8^+^ T cells accumulate in tumor, being mostly central memory T cells (CD44^hi^CD62L^hi^) -No significant change of CD3^+^ T cells in spleen -Elevated serum levels of TNF-α and IFN-ƴ -Restore immune balance to healthy state and prolong relapse-free survival	B16 metastatic melanoma	C57BL6j mice	Topical ALA 20% *w/w*, loaded in CDG2/HA-contructed nanoparticles	25 mW/cm^2^, 5 min	[60]

In conclusion, the systemic and long-term immune effect of PDT relies largely on adaptive immunity, and data have shed light on the role of T lymphocytes, with a focus on CD8^+^ T cells (as exemplified in Table 2). The importance of humoral adaptive immunity has, however, not been thoroughly demonstrated, although a study has shown that expression of C3a receptor increased on B lymphocytes after Photofrin-PDT, while its expression remained unchanged on T lymphocytes [24]. Since CD8^+^ T lymphocytes play an essential role in antitumor responses, the mechanism of how PDT induces activation of these cells would therefore be of prime interest in future research.

## 4. Clinical Evidence of Antitumor Immunity Induced by PDT

Although Photofrin and ALA have been used in numerous clinical trials, only a small number of these have investigated the immune responses of PDT. Most clinical data derive from studies applying topical ALA-PDT to patients with basal cell carcinoma (BCC), while fewer studies investigate this aspect of PDT in the case of MAL (the methyl ester of ALA), Photofrin, or Temoporfin (Table 3).

### 4.1. Acute Immune Response

Acute immune responses are confirmed in BCC patients treated with topical ALA- or MAL-PDT. Within 1 h after PDT, increased infiltration of neutrophils is already visible at the tumor site, reaching its peak at 4 h, and declining to basal level after 48 h [62]. Interestingly, activity of neutrophils, as indicated by chemiluminescence, was found upregulated in peripheral blood 4 h after PDT [63], indicating this acute immune response might not be restricted to the treated lesion. In addition, after 48–72 h myeloid cells such as macrophages and mast cells accumulate at the treated site [62]. Increased expression of immunostimulatory IL-23, IL-22, IL-17, and IFN-γ was found in peritumoral inflammatory cells shortly after PDT [64]. Moreover, serum levels of a immunosuppressive cytokine TGF-β were shown to decline at 4 h after PDT [63]. Together, these clinical data support the stimulatory effect of PDT on acute immune responses, which is consistent with the observations from preclinical studies [22,23,24,51,52]. However, these responses seem to maintain within a week, after which they tend to decline, and become non-detectable after one month [64,65,66], suggesting a transient inflammatory response following PDT.

Likewise, increase of inflammation was also detected shortly after Photofrin-PDT, as indicated by elevated levels of serum IL-6, a pro-inflammatory cytokine, in patients with esophageal squamous cell carcinoma (ESCC) at one week after treatment, but this declined soon after two weeks [67]. In addition, a slight increase of peripheral neutrophils and monocytes was observed in these patients at one week. However, the late time point chosen in this study might not provide clear evidence of the initial rapid inflammation. Another interesting study involved Temoporfin-PDT in head and neck squamous cell carcinoma (HNSCC), where increased serum levels of IL-6 were detected with a peak at 24 h, and HMGB1 with a peak at one week after treatment [68], further supporting PDT as a potent inducer of acute inflammation.

### 4.2. Adaptive Immune Response

Being potent APCs, DCs act as a central link with the adaptive immune response. Clinical data on DCs in PDT-induced immune responses are scarce, and mainly come from studies of BCC patients, where Langerhans cells were analyzed. Longo et al. found increase of Langerhans cells, accompanied with lymphocyte-like cells, at the tumor site one week after ALA-PDT [65]. In contrast, Evangelou et al. reported decrease of Langerhans cells at the treated site at 1 and 24 h after MAL-PDT [69]. This discrepancy could be explained by the difference in time point, treatment protocol, or way of measurement chosen in the study. On the other hand, the decrease of Langerhans within 24 h might be due to their migration from the tumor site to lymphoid organs, where they present antigens and subsequently activate T cell responses. Obviously, further study is warranted to elucidate this proposed mechanism. 

**Table 3:** Clinical studies investigating the immune response to PDT in cancer treatment.

Disease (Stage/Subtypes)	PS/Dose	Illumination Protocol	No. of Patients	* Prior Treatment	Immune Events and Time Points Post PDT	Samples	Ref
**BCC (superficial and nodular BCC)**	Topical ALA (10% emulsion)	75 J/cm^2^ 70–126 mW/cm^2^	10	N/A	-Increase of Langerhans cells associated with lymphocytes in tumor at 1 week -No sign of immune cells in tumor at 4 weeks	Tumor	[65]
**BCC (superficial BCC)**	Topical ALA (20% emulsion)	100 J/cm^2^ for 10 min	15	NA	-Neutrophils increase at 4 h, and declines to basal levels after 48 h -Mast cells tend to increase up to 72 h -Lymphocytes increase at 24 h -Macrophages continuously increase at 48 and 72 h	Tumor biopsy	[62]
**BCC (ulcerating, superficial and nodular BCC)**	Topical ALA (10% emulsion)	100 J/cm^2^ ≤150 mW/cm^2^	17	Yes (only surgical excision)	-Increased neutrophil activity in blood at 4 h -Decreased expression of IL-1β by lymphocytes at 4 h -No significant changes in IL-6, IL-2 and TNF-α by lymphocytes -Decreased TGF-β1 levels in serum at 4 h	Peripheral blood; Serum	[63]
**BCC (superficial and nodular BCC)**	Topical ALA (20% emulsion) or Photofrin i.v. (1 mg/kg)	100–260 J/cm^2^ 150 mW/cm^2^	21	Yes, 12 patients	-Increased tumor antigen-specific T cell response at 1 and 2 weeks -Anecdotal regression of lesions outside the treated area (ALA)	Peripheral blood	[70]
**BCC (superficial and nodular BCC)**	Topical MAL	37 J/cm^2^ for 7 min 40 s (2 sections with 1-week interval)	10	No	30 min to 2 h: -Increase of peritumoral inflammatory cells -Increased levels of IL-23, IFN-γ, IL-22 and IL-17 1–12 weeks: -Decrease of peritumoral inflammatory cells -Decreased levels of IL-23, IFN-γ, IL-22, and IL-17	Tumor biopsy	[64]
**BCC (superficial BCC)**	Topical MAL (2 g)	37 J/cm^2^, 70 mW/cm^2^	8	N/A	-Increased infiltration of neutrophils at 1 and 24 h -Increased expression of E-selectin in blood vessels at 1 and 24 h -Decrease of Langerhans cell numbers at 1 and 24 h	Tumor biopsy	[69]
**VIN (high-grade)**	Topical ALA (20% emulsion)	50–100 J/cm^2^	32	Yes, 6 patients	-Loss of HLA class I in PDT nonresponders -Increase of CD8 T cell infiltration in PDT responders compared with nonresponders	Tumor biopsy	[71]
**VIN (high-grade)**	Topical MAL	50 J/cm^2^ (2 sections with a month interval)	11	Yes	-No statistically significant differences in CD4, CD8, CD1a, and CD68 cells was detected at 26 w	Tumor biopsy	[66]
**ESCC (early stages)**	Photofrin (1 mg/kg)	80 J/cm^2^	8	N/A	-Increased of peripheral granulocyte at 1 and 2 weeks -increase of peripheral monocyte at 1 week -Increased levels of IL-6 in serum at 1 week -Increased number of peripheral Treg at 2 weeks -Inhibited immunosuppressive function of peripheral Treg at 2 w -No significant change of peripheral lymphocyte numbers or systemic CD4^+^ T cell numbers at 1 week -No significant changes of tumor-infiltrating Treg at 1 and 2 weeks	Peripheral blood; Serum; Tumor biopsy	[67]
**HNSCC (Stage 1–4)**	Temoporfin (Foscan)	N/A	9	Yes	24 h, 1 week, and 4–6 weeks: -Increased frequency of NK cells at 4–6 weeks -Increased frequency of Treg up to 6 weeks -Increased concentration of HMGB1 (peak at 1 week), IL-6 (peak at 24 h), and IL-10 (peak at 24 h) -Decreased concentration of Perforin (lowest at 24 h and 1 week)	Peripheral blood; Serum	[68]

PDT, photodynamic therapy; BCC, basal cell carcinoma; ESCC, esophageal squamous cell carcinoma; VIN, vulva intraepithelial neoplasia; CIN, cervical intraepithelial neoplasia; HNSCC, head and neck squamous cell carcinoma; Treg, regulatory T cells; NK: natural killer; IL, interleukin; TNF-α, tumor necrosis factor alpha; TGF-β, transforming growth factor beta; IFN, interferon; HMGB, high-mobility group protein; ALA, aminolevulinic acid; MAL, methyl aminolevulinate; N/A, non-available. * Previous adjuvant or radio (chemo) therapy or surgery.

The involvement of lymphocytes is supported by studies from BCC patients receiving ALA- or Photofrin-PDT. Increased lymphocytes at the tumor site were detected 24 h after ALA-PDT, and remained elevated for at least 72 h, being CD4^+^ more abundant than CD8^+^ lymphocytes [62]. In another study, activity of peripheral lymphocyte activity was analyzed at 4 h after PDT, in which cellular expression of IL-1β, IL-6, IL-2, and TNF-α was measured [63]. Nevertheless, only a slight decrease of IL-1β was detected, suggesting 4 h might not be the optimal time point to measure lymphocytes in peripheral blood. Of note, both Photofrin- and ALA-PDT enhanced lymphocyte recognition of a TAA hedgehog-interacting protein (Hip1) expressed in BCC [70]. Antigen recognition was significantly greater in patients whose lesions were treated with PDT, in comparison to surgical removal. The antigen-specific immune response described in this study is, most likely, mediated by CD8^+^ T cells since Hip1 peptide forms complexes with MHC-I molecules. In supporting this, a study from patients with vulva intraepithelial neoplasia (VIN) (*n* = 32) treated with ALA-PDT showed that VIN that display loss of MHC class I (*n* = 9) failed to respond to the treatment, whereas the responders exhibited significantly higher CD8^+^ T cell infiltration than non-responders [71]. In addition to T helper and cytotoxic lymphocytes, increasing number of regulatory T lymphocytes (Treg) were also observed in peripheral blood of patients receiving PDT treatments [67,68].

### 4.3. Systemic Immune Response

Even though PDT is a treatment applied locally in cancer patients, available clinical data suggest its potential to trigger systemic immune responses, and in some cases even an abscopal effect. For instance, remission of tumors outside the treated area has been reported in several cases of BCC [70] or angiosarcoma [72], following the local treatment with ALA- or Fotolon-PDT, respectively. In the former study, the authors described that such effect was accompanied by an increased cytolytic activity of splenocytes and infiltration of CD8^+^ lymphocytes in untreated tumors [70]. Besides, supporting evidence also includes enhanced activity of immune cells in peripheral blood after local treatments of PDT, such as neutrophil [63] and lymphocyte activity [62,70] (see Section 3.1.1 and Section 3.1.2). In addition, NK cell numbers were found increased in peripheral blood of HNSCC after Temoporfin-PDT [68]. Treg isolated from peripheral blood exhibited reduced immunosuppressive activities in ESCC patients after Photofrin-PDT [67]. These clinical data are however scarce. As such, obtaining more evidence will contribute to a better understanding for such potential of PDT, and to ultimately being able to use the information for improving therapeutic outcomes.

## 5. Potentiating PDT with Immune Modulation

Despite much evidence showing immune stimulation after PDT, the generation of robust antitumor immune responses triggered by PDT is, however, not often the case [73]. This could be, at least partly, explained by the fact that tumors are heterogenous and exhibit different immunogenicity reflected by more or less immune cell infiltrates (also referred to as “hot” versus “cold” tumors). Another hurdle are loads of immunosuppressive factors present locally at the tumor site or systemically [74], which occurs often in advanced cancer patients [75]. Strategies by combining agents that boost the immune system and/or reverse the immunosuppression would, therefore, enhance the occurrence of effective and long-lasting immune responses against cancer, at the same time as PDT destroys the actual tumor. These include, but not limited to, various immunostimulants, blocking or depleting immunosuppressive (cellular) factors, inducing tumor antigens and immune-potentiating vaccines such as DC-based vaccines.

### 5.1. Immunostimulants

Being widely used as adjuvants for enhancing cancer vaccines, TLR agonists, such as Bacillus Calmette–Guérin (BCG, TLR-2/4), imiquimod (TLR-7), and CpG oligodeoxynucleotide (CpG ODN, TLR-9), are potent immune stimulants [76]. Through binding to PRRs on immune cells, they can improve antigen delivery, processing, and presentation by APCs, or induce immunomodulatory cytokines production [76]. It has been shown that administration of BCG increased the number of tumor-free mice after PDT, regardless of the type of PS employed, including Photofrin, benzoporphyrin derivative, Temoporfin, mono-L-aspartyl-chlorin e6, lutetium texaphyrin, or zinc phthalocyanine [31]. Interestingly, the ratio of memory T lymphocyte subsets is further increased at tumor lymph nodes in the combination with BCG, compared to Photofrin-PDT alone. The use of CpG ODN in conjunction with PDT has also been successfully demonstrated. For instance, the co-injection of CpG with Radachlorin-PDT-generated tumor lysates elicited a strong antitumor immune response, resulting in increased production of tumor-specific antibodies and cytotoxic T cell responses [77]. Besides, Verteporfin-PDT in combination with CpG demonstrated decreased tumor sizes and better survivals, compared to either treatment alone [78]. Topical PDT, generally applied to treat cancer limited to the skin surface, when co-applied with imiquimod cream, has been proved effective as well for invasive squamous cell carcinoma, in both mice models and humans [79]. Zymosan, being known as a TLR-2 agonist, has been shown to augment the cure rate of tumor after Photofrin-PDT, as well as the levels of C3 complement [80]. Other TLR agonists such as mycobacterium cell wall extract (MCWE) also demonstrated significant synergetic effects on cancer treatment with PDT using different PSs [81]. Recently, there has emerged a new class of immunoadjuvants, e.g., N-dihydrogalactochitosan—a semisynthetic cationic carbohydrate polymer and derivative of chitin (an abundant natural polysaccharide) exhibiting complex advantageous properties encompassing both immunostimulatory effectiveness and amplifying PDT-mediated direct lethal cell kill [82].

A major sign for tumor ICD is the release of DAMPs, such as CRT, HSP70, HMGB1, or ATP, which through binding to PRRs can induce immune maturation and activation at the local site (as discussed in Section 3.1.3). In fact, recombinant CRT, upon peritumoral injection, has been shown to boost the therapeutic effect of PDT and/or PDT-generated cancer vaccines [83]. In addition, immunostimulants such as glycated chitosan [84], vitamin D3-binding protein-derived macrophage-activating factor (DBPMAF) [85], neutrophil promoting factor G-CSF [34,86], and Schizophillan (SPG) [87] have shown beneficial effects in the case of Photofrin-PDT, and CCL8 (monocyte chemoattractant protein-2) in the case of ALA-PDT [88].

### 5.2. Blocking or Depleting Immunosuppressive (Cellular) Factors

Tumors that are resistant to first-line therapy (surgery, radiotherapy, and chemotherapy), or in advanced stages, often develop severe immunosuppressive burden. This is featured by upregulation of inhibitory molecules that restrain immune activation, signals that promote tumor growth and/or accumulation of immunosuppressive cells. Therefore, approaches that reduce/inhibit the immunosuppressive factors and restore the immune responses would, at large, improve the therapeutic outcomes of PDT in these cancers. An excellent example of this is the addition of immune checkpoint inhibitors (e.g., anti-PD-L1 and anti-CTLA-4), which have revolutionized the treatment of various cancers. The blockade of PD-L1 or CTLA-4 restores the tumoricidal activity of lymphocytes, and, more recently, has also been shown to synergize the therapeutic effect of PDT, where abscopal effects were observed [89,90,91,92]. Interestingly, this synergistic effect is also observed with third-generation photosensitizers, i.e., antibody–photosensitizer conjugates for targeted PDT, resulting in increased tumor infiltration of mature DCs and T lymphocytes, abscopal antitumor effects, and immunologic memory [93,94]. Another possible mechanism of PDT resistance is the accumulation of immunosuppressive cells at the tumor sites, including Tregs and myeloid derived suppressive cells (MDSCs). These cells, through heterogenous mechanisms, exhibit a potent ability to inhibit several components and phases of immune responses [95,96]. Upon selective depletion of Tregs with low dose cyclophosphamide prior to PDT, a dramatically decreased tumor size and increased survival was observed [97]. In addition, depletion of MDSCs by using GR1 blocking antibody improved tumor cure rates of PDT. Such beneficial effect, nevertheless, disappeared when anti-GR1 was injected immediately, instead of 1 h, after PDT illumination [98]. The abrogated therapeutic effect could be caused by the unwanted depletion of neutrophils, with anti-GR1 at the acute phase of PDT (i.e., within an hour), where neutrophils play an essential role in stimulating immune responses. In addition to cellular fractions, soluble mediators such as TGF-β and PGE2 also contribute to the immunosuppressive tumor microenvironment [99]. As such, agents that inhibit these factors would also be beneficial to enhance the anti-tumor immune responses induced by PDT.

### 5.3. Importance of Tumor Antigens

Tumor antigens can be unique tumor-specific antigens [100], antigens that can be found in both normal and tumor tissues but are overexpressed in tumors [101], or antigens of viral etiology [102]. Recognition of tumor antigens is the first step for establishing long-term immune memory in cancer immunotherapy. As such, the type and degree of tumor antigen expression plays an essential role in the immune effects of PDT. It has been suggested that PDT-mediated ER stress and ROS production are likely to increase the expression and liberation of antigens [73], but it has never been investigated. In reality, most clinical tumors, however, exhibit weak immunogenicity and therefore limit the long-term effects of PDT. In addition, tumor cells may escape immune surveillance by losing the expression of tumor antigen or downregulation of MHC I molecules. Restoring or inducing antigen expression or presentation by the tumor cells is, therefore, key to tackling this type of tumors. Attempts have been made to induce expression of a silenced tumor antigen P1A with 5-Aza-2′-deoxycytidine (a methyltransferase inhibitor) in four different tumor models (Lewis lung carcinoma, 4T1 mammary carcinoma, CT26 colon carcinoma, and EMT6 mammary carcinoma) that are treated with Photofrin-PDT. Of particular interest, the combination strategy leads to complete tumor cures and long-term survival in CT26 and EMT6 models, and even resistance to the re-challenge with the same tumor cells [59]. Furthermore, these effects were largely dependent on CD8^+^ T lymphocytes.

### 5.4. Immune Potentiating Therapeutic Vaccines

Therapeutic vaccines against cancer are getting more attention since (neo)antigen identification has become more technically feasible in a time frame that allows detailed molecular analysis of the specific peptide sequences [103]. Several types of vaccines have been explored as well as combined with PDT. The use of DC-based vaccines for cancer has been extensively investigated, with more than 200 complete clinical trials to date [104]. Preclinical evidence demonstrate that PDT-treated tumors enhance DC recruitment, maturation, and cytokine secretion [45,54,105,106,107]. Indeed, using PDT-treated tumor cells as adjuvant for DC-based vaccine (PDT-DC vaccine) has been proved to trigger stronger protection against tumors [12,108,109], and enhanced T lymphocyte responses [109], compared to using freeze/thaw-treated tumor cells. Furthermore, local PDT followed by intratumoral injection of DC [10,110] has shown decreased tumor sizes, better survival, and higher cytotoxicity mediated by CD8^+^ T lymphocytes and NK cells [10], compared to either group alone. Using CT26 colorectal carcinoma and B16 melanoma mice models, Saji at al. showed this strategy significantly enhanced tumor-cured rates in mice and prolonged the survival of mice of which the tumors were not cured [61]. Remarkably, this strategy induced regression of tumor at distant sites including lung metastases, implying the systemic antitumor effect of PDT could be greatly enhanced by such strategy.

Molecularly defined therapeutic peptide vaccination has been successfully combined with chlorin e6-based PDT in mouse models and convincingly shows synergistic clearance of primary tumors. Abscopal CD8 T cell-mediated effects were shown by reduction of secondary tumors [111]. These vaccines have been even further improved by conjugation to a defined DC-stimulatory TLR2 ligand, which showed strong anti-tumor effects when combined with PDT [112].

In conclusion, a better understanding of the tumor microenvironment and the development of current immunotherapies have provided a wealth of opportunities for devising combination strategies to trigger robust immune responses after PDT. For instance, approaches that reverse hypoxia at the tumor site [113] would likely enhance the PDT-induced immune responses, since severe hypoxia contributes to the tumor immunosuppression, and also adequate oxygen levels are critical for PDT efficacy. However, human bodies are far more complicated and heterogenous than laboratory models. Therefore, efforts are ongoing towards a better preclinical model as close as possible to patients, such as humanized mouse models engrafted with patient-derived cancer organoids and immune system. Besides, the PDT parameters such as choice of PS, doses of both PS and illumination, fluence rate, and drug–light interval are also important in optimizing the immune responses.

## 6. Future Directions for Clinical Research

Preclinical studies point at PDT as very effective in breaking the immunotolerance in treated tumors, overturning the immunosuppressive tumor microenvironment, and instigating the development of a strong adaptive immune response against these lesions. However, scarce information has been provided concerning these events in the clinical setting (see Table 3 for an overview of the reviewed clinical studies). Analysis of the literature in this case is limited due to the low number of clinical studies. With this in mind, suggestions are given in this section that could contribute to the collection of additional and valuable information from future clinical studies, as well as promote further understanding and exploration of the implication of the immune system for the greater success of the PDT treatment.

### 6.1. Recommendations for Immune Monitoring

**Figure 2:**
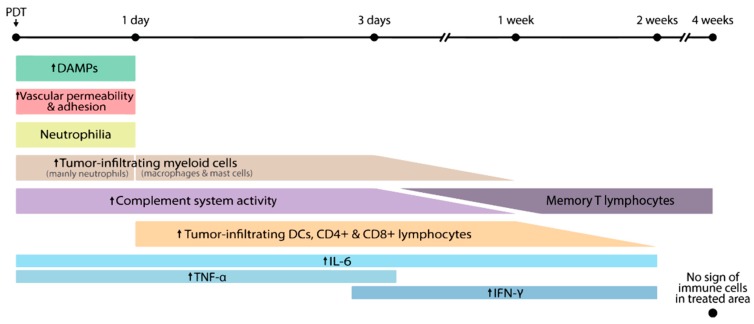
Indicative timeline of the main immunological events developed after PDT. Shortly after PDT, an initial inflammatory response arises due to the exposure of DAMPs and complement activation, and is facilitated by the increased vascular adhesion and permeabilization at the tumor site during the first 24 h post-PDT. A pronounced neutrophilia occurs, which is followed by increased numbers of tumor-infiltrating neutrophils during the first 24 h, and infiltrating macrophages and mast cells at least during the first 72 h. One day post-PDT, DCs and lymphocytes start accumulating at the tumor site. This triggered adaptive response might last for two weeks and is accompanied by high levels of IL-6, TNF-α, and IFN-γ, as well as elevated complement activity. The initial immunological response is transient and four weeks post-PDT immune cells are no longer detected in the treated area. After this initial response, tumor-specific memory effector T cells can be expected to be present in circulation or in the tumor-draining lymphoid organs.

Proper immune monitoring of the initial innate effects, as well as the downstream effects on the adaptive response, is key to designing successful combination strategies. Numerous clinical trials focus on the efficacy of the PDT treatment, but little can be found on its effects on the immune system. In general, the available clinical evidence seems to correlate with preclinical data, but this evidence is limited, and further study is needed to safely claim correlation. If we are to learn from preclinical studies, most significant changes in cells and mediators occur during the first day (innate response) and first week (adaptive response) after PDT. Based on the reviewed preclinical and clinical data, a timeline is shown in Figure 2 as an indication of the main immunological events developed post-PDT. This timeline might serve as guidance when designing new clinical studies aiming to further characterize the immune response after PDT.

Human studies could first focus on the confirmation of the most determinant immune events observed in animals. Certain cytokines, i.e., IFN-γ, TNF-α, and IL-6, have been found to play important roles in preclinical studies, particularly during the initial inflammation and the transition to a T cell-mediated response during the first week post-PDT [29,51]. Special attention should be paid to IL-6 due to its tumor promoting role observed in mice under particular PDT regimens [114,115]. In fact, high levels of IL-6 have been reported within the first two weeks after clinical PDT [67]. As for the cellular components mediating the immune response, a marked neutrophilia [34], maturation of DCs [51], elevated numbers of T cells [116], and increased cytotoxic activity of CD8^+^ T cells [59] are features observed after preclinical PDT. Therefore, detection and study of these cell types would be a logical starting point in patients.

The effects of PDT on the adaptive response, especially tumor-specific T cell expansions which are observed in preclinical models in lymphoid organs and in blood, allows monitoring of patients by collecting blood samples before and after therapy. Sustainment of tumor-specific effector memory T cells is important for cures and systemic control of recurrences; thus, follow-up blood sampling is recommended. Furthermore, as cancer type related epitope knowledge and identification of new tumor-specific T cell specificities will be increasing in the near future, monitoring of tumor-specific T cell responses by high-end flow cytometry will become feasible. Modern technologies to identify patient-specific neo-epitopes by exome sequencing and HLA-binding algorithms will become more rapid and applicable for individual patient monitoring. Moreover, quantitative analysis of the levels of circulating specific T cells but also the monitoring of the quality of the T cell response by analysis of cytokine profiles and cell surface markers for effector, memory, or regulatory T cell subpopulations are essential. This information could decisively influence further T cell directed treatment options to be combined with PDT (e.g., specific stimulation with agonistic antibodies or stimulatory vaccines). In addition, following levels of other immune cell types which have remained relatively unexplored in animals, such as NK cells, B cells, and Treg cells, would allow further characterization of the response. Once the critical immune elements in humans have been characterized, and when necessary, immunomodulatory agents can be specifically and wisely applied in combination with PDT to maximize efficacy of the treatment. 

While preclinical studies allow a broad use of techniques to modify the animal in order to characterize a particular process in detail, this is limited in the case of humans. Non-invasive methods in the clinic for detection and measurement of the immune response after PDT are of extreme value, and development of such techniques would greatly add to the understanding of this process. The obvious current choice is the collection of blood for systemic analysis, as highlighted above, and some studies point at reflectance confocal microscopy for examining the tumor site in the case of superficial skin cancers [65]. This imaging tool allows monitoring of the tumor response as well as detection of immune cells at the tumor over time. The activity of the different immune cells isolated from patients can be evaluated ex vivo and correlated with changes in other cellular or soluble components, as well as with the clinical outcome in order to be able to make predictions based on correlations. It is also of interest to study the response of cancer lesions outside the treated area. This is an unexplored event in the clinic (with exception of case reports), relatively easy to monitor and of great clinical value. Study of the immune response as here described can be performed as secondary endpoints in clinical trials focused on PDT safety and efficacy.

### 6.2. Recommendations for Optimizing PDT Outcome

PDT is now firmly stablished as able to render an anti-tumor immune response; however, this does not necessarily equal to tumor cure. To achieve this, it has to be ensured that the elicited immune response remains vigorous and durable. Future preclinical and clinical research has to optimize means of preventing that the elicited immune response remains incomplete and stalls before the immune destruction of both local and distant tumor deposits is complete. To meet this goal, we review here the use of a series of PDT adjuvants and joint agents based on their potential for preventing immunoregulatory mechanisms known to hinder the effectiveness of PDT. We strongly believe that further research along these lines can certainly boost the potential of PDT to its maximum. While considerable advances were made in developing and characterizing PDT-generated cancer vaccines in preclinical studies, very little effort has been made in translating this into the clinic and establishing the benefits of PDT vaccines with cancer patients. It seems likely that clinical results can turn out to be even more positive than predicated with fast growing animal models, especially with (thus far untested) full scale multiple vaccination regimens and combination with adjuvants preventing development of immunosuppressive microenvironment.

Preclinical as well as clinical evidence indicates that the illumination conditions have an important impact on the activation of the immune system and overall success of the PDT therapy [70,117,118,119]. It has been suggested that high fluence rates deplete oxygen levels at the tumor site in patients with BCC [119], resulting in a possibly inefficient treatment although still effective due to the extensive vascular and tumor damage. Moreover, high fluences seem to have a negative impact on immune reactivity towards tumor antigens in patients with BCC [70], supporting the fact that high fluences may not be ideal for the development of an immune response, likely due to a potent vascular shutdown that prevents immune cells from reaching the tumor area. On the other hand, preclinical studies point to the fact that low fluences with low fluence rates inflict diffuse damage and facilitate the enhancement of an inflammatory response, but may yield lower cure rates [120].

The illumination protocols commonly used in the clinic are, in general, devised to induce extensive damage to the primary tumor, while the immunobiology of PDT is overlooked. Based on the reviewed clinical studies, immunostimulatory PDT regimens consist of moderate to high fluences, low fluence rates, and treatment of small surface areas. These illumination regimens might be especially beneficial when combined in the clinic with immunostimulatory strategies. Interestingly, a two-fold fractionated illumination scheme (low fluence followed by high fluence) has shown superior efficacy than single illumination to treat patients with superficial BCC [121]. This suggests great clinical benefit can arise from the design of two-step PDT programs consisting of a first immune-stimulating illumination regimen, followed by a regimen that mediates potent tumor destruction, which has proven favorable in the preclinical setting [117].

All things considered, further preclinical exploration of the different PDT regimens is still essential. Due to the critical role of the illumination parameters, we would like to underline the importance of these being always specified in published studies. Likewise, we also highlight the importance of stratifying patients based on previous received treatments, such as radio-, chemo- and adjuvant therapy. These, upon repeated use, can have a large effect on the immune system, leading to exhausted immune responses or immunosuppression. To our surprise, four out of the 10 reviewed clinical studies have not addressed this aspect, and only three of these 10 gave additional details regarding prior treatments.

## 7. Conclusions

Many studies here reviewed document the activation of the immune system post-oncologic PDT. Preclinical studies point at PDT as very effective in breaking the immunotolerance in treated tumors, disturbing the immunosuppressive tumor microenvironment and developing a strong and systemic adaptive immune response against even distant tumor lesions. Clinical evidence, however, is unfortunately very scarce. We believe that collecting more of this evidence in the clinical setting will contribute to the full understanding of these events in humans. Consequently, this would enable the design of new trials in which the immunological potential of PDT is explored in its full, for more effective treatment of many cancer patients.

## Figures and Tables

**Table 1:** Principal characteristics of the photosensitizers Photofrin and ALA.

Photosensitizer (PS)	Porfimer Sodium	ALA
Trade name	Photofrin	Levulan
Composition	Mixture of hematoporphyrin derivatives	Heme precursor (prodrug) converted to Protoporphyrin IX
Maximum absorption	630 nm	630–635 nm
Adsorption at maximum wavelength	3000 M^−1^cm^−1^ (weak)	5000 M^−1^cm^−1^ (weak)
Administration	Systemic (i.v.)	Systemic (i.v.), oral, topical
Time of illumination after PS administration	40–50 h	Within 24 h
Clinical dose	1 mg/kg	10–20% ALA emulsion (topical)
Illumination conditions	80–260 J/cm^2^ ≤150 mW/cm^2^	75–260 J/cm^2^ ≤150 mW/cm^2^
Singlet oxygen quantum yield	0.89 (high)	0.56 (moderate)
PS localization	Mitochondria	Cell membrane, mitochondria, lysosome
Induced cell death	Apoptosis (mainly) *	Apoptosis (mainly) *
Disadvantages	Limited tissue penetration Skin photosensitization (6 weeks)	Limited tissue penetration Moderate pain if skin treatment
Approved indications	Bladder, esophageal, skin, and non-small cell lung and cancer	Actinic keratosis and other non-oncologic indications
Ongoing clinical trials	Brain, cervical, breast and head and neck cancer, among others	Basal cell carcinoma, cervical neoplasia, and head and neck cancer, among others

* Apoptosis is the main mechanism reported in vitro, although necrosis is often described in vivo.
